# Clinical Outcome in Singleton and Multiple Pregnancies with Placental Chorangioma

**DOI:** 10.1371/journal.pone.0166562

**Published:** 2016-11-11

**Authors:** Meeli Sirotkina, Konstantinos Douroudis, Nikos Papadogiannakis, Magnus Westgren

**Affiliations:** 1 Section of Perinatal Pathology, Department of Pathology, Karolinska University Hospital Huddinge, Stockholm, Sweden; 2 Department of Laboratory Medicine, Division of Pathology, Karolinska Institutet, Stockholm, Sweden; 3 Department of Neurology, Haukeland University Hospital, Bergen, Norway; 4 Department of Clinical Medicine, University of Bergen, Bergen, Norway; 5 Department of Clinical Science, Intervention and Technology, Division of Obstetrics and Gynecology, Karolinska Institutet, Stockholm, Sweden; 6 Department of Obstetrics and Gynecology, Karolinska University Hospital, Stockholm, Sweden; Shanghai Jiao Tong University, CHINA

## Abstract

**Introduction:**

Chorangiomas (CAs) are the most common non-trophoblastic tumor-like-lesions of the placenta. Although the clinical significance of small CAs is unknown, the large lesions are often associated with maternal and fetal complications. The aim of our study was to assess the maternal clinical characteristics and neonatal outcome in singleton and multiple pregnancies with placental CA.

**Materials and Methods:**

Among 15742 selected placentas 170 CAs were diagnosed. Pregnancy and neonatal outcomes were analyzed in singleton (n = 121) and multiple (n = 49) pregnancy groups including 121 and 100 neonates, respectively.

**Results:**

The frequency of APGAR score <7 at 5 minutes (p = 0,012), abnormal pulsatility index (p = 0,034), and abnormal blood flow class (p = 0,011) were significantly higher in neonates from singleton compared to multiple pregnancies. Significantly smaller CAs in singleton pregnancies were related to small for gestational age neonates (p = 0,00040) and neonates admitted to the neonatal care unit (p = 0,028). In singleton pregnancies, significantly smaller CAs were associated to maternal preeclampsia (p = 0,039) and larger CAs to multiparity (p = 0,005) and smoking (p = 0,001) groups. The frequency of preeclampsia was high in both singleton and multiple pregnancy groups (41,32% vs 26,53%, respectively), however, the difference did not reach the level of statistical significance.

**Discussion:**

A high incidence of preeclampsia in cohort of placental CA might lead to a possible recognition of CAs as potential morphologic indicator of placental hypoxia.

**Conclusion:**

A more favorable pregnancy outcome in multiple gestations compared to the singleton gestations with CAs might reflect an adaptive mechanism for increased demand of oxygen and associated placental tissue hypoxia in this group.

## Introduction

Chorangiomas (CAs) are the most common non-trophoblastic tumor-like-lesions of the placenta, occurring in approximately 1% of pregnancies [1]. In majority of cases, they are small or microscopic, and are found only after careful morphologic examination of the placenta. The clinical significance of microscopic CAs remains unknown. Large lesions, however, more than 4 cm in diameter, are rare in obstetric practice and can be diagnosed by prenatal ultrasound imaging or on routine pathologic examination. Large CAs are often assotiated to chronic arterio-venous shunting within the placenta and linked to a number of pregnancy complications including fetal anemia, fetal hydrops, polyhydramnios, intrauterine fetal growth restriction (IUGR) and increased perinatal mortality [2]. Further, the presence of CAs is related to an increased maternal age, multiple gestations, preeclampsia and HELLP (hemolytic anaemia, elevated liver enzymes and low platelet count) syndrome [2]. The aim of our study was to assess the maternal clinical characteristics and neonatal outcome in singleton and multiple pregnancies with placental CA and to elucidate the clinical significance of these tumors.

## Materials and Methods

During the period of 1996–2012, 15742 placentas, including 2112 (13,4%) from multiple pregnancies (2095 twin placentas and 17 triplet placentas), were examined at the Pathology Department of Karolinska University Hospital, Stockholm, Sweden. Regional consensus indications for pathological examination of the placenta included prematurity <32 weeks, preeclampsia including HELLP syndrome, repeated hemorrhage, abruption, fetal/neonatal asphyxia (Apgar <7 at 5 min and/or umbilical artery pH <7,0), non-immune hydrops, IUGR, fetal or perinatal death, macroscopically abnormal placenta or umbilical cord and suspicion of chorioamnionitis. Twin placentas were referred in cases of complicated pregnancy (prematurity, twin to twin transfusion syndrome and IUGR), whereas chorionicity alone was clearly not an indication for referral.

CAs were diagnosed according to the morphological criteria as nodular lesions of capillary vascular channels surrounded by trophoblast [3] in 170 cases (121 singleton and 49 multiple pregnancies). The lesions were coded as hemangiomas according to the Systematized Nomenclature of Medicine (SNOMED). The current study follows up the population of placentas whose morphological characteristics we have been previously described by Sirotkina et al [2] and further examines for the first time the maternal clinical characteristics and the neonatal outcome in singleton and multiple pregnancies with placental CA.

Maternal baseline characteristics, pregnancy and neonatal outcome data were retrieved from original medical records or electronical databases (Obstetrix, TakeCare). Gestational age was evaluated according to ultrasound examinations performed in the beginning of the second trimester.

Maternal characteristics of 170 mothers with placental hemangioma and neonatal outcomes of related 221 infants (121 from singleton pregnancy and 100 from multiple pregnancies) were analyzed in two groups, singleton and multiple pregnancies. Further, the characteristic were analyzed according to the diameter of the CA (N = 169; in one case CA was weighted but not measured). Maternal body mass index (BMI) ≥ 25 was considered as overweight and prematurity was defined as delivery before the 37th week of gestation. Birth weight adequacy to gestational age was evaluated according to the regional growth charts. Fetal and placental circulation was monitored with Doppler ultrasound by recording flow velocity signals from the umbilical artery in a free loop of the umbilical cord. Doppler velocimetry was performed transabdominally. All recordings were performed during periods of absence of fetal breathing and movements, and during voluntary maternal apnea. The umbilical artery PI was calculated automatically by the ultrasound systems according to the method of Gosling et al. [4]. A pulsatility index (PI) > mean + 2 SD was classified as abnormal. The fetal umbilical artery Doppler velocimetry was assessed by a semi-quantitative method, resulting in four blood flow classes (BFC): BFC 0 –positive diastolic flow, PI ≤ mean + 2 SD; BFC I—positive diastolic flow, PI > mean + 2 SD and PI ≤ mean + 3 SD; BFC II—positive diastolic flow, PI > mean + 3 SD and/or absent end diastolic flow; BFC III—absent end diastolic flow and/or reversed diastolic flow. In the present study, normal fetal umbilical artery blood flow velocity is classified as BFC 0 and abnormal as BFC I–III [5, 6]. The Regional Ethical Review Board in Stockholm approved the study.

### Statistical analysis

Statistical analysis performed using the R software (version 3.0.3). Pearson’s Chi-squared, Fisher’s exact, Wilcoxon rank-sum and Kruskal-Wallis test were used where appropriate, and logistic regression analysis applied to estimate odds ratio (OR) values with 95% confidence intervals (CI). A p value of <0.05 was considered significant for all analyses.

## Results

The incidence of CAs within our cohort (N = 15742; 1.08%) was significantly higher in the group of multiple pregnancies compared to the singleton group (2.32% vs 0.89%, respectively, p<0.0001). The multiple gestation group included 29 dichorionic/diamniotic-, 15 monochorionic/diamniotic-, 3 monochorionic/monoamniotic- and 2 trichorionic/triamniotic placentas. The baseline maternal characteristics in singleton and multiple pregnancies showed no statistically significant differences between the two studied groups (Table 1). The frequencies of preeclampsia (41.32% vs 26.13%) and the incidence of diabetes (6.67% vs 2.04%) were higher in the group of singleton compared to the multiple pregnancies group, respectively; however, they did not reach the level of statistical significance.

**Table 1:** Maternal baseline characteristics and pregnancy outcomes in singleton and multiple pregnancies.

	Singleton	Multiple	P value
	(N = 121)	(N = 49)	
**Maternal age**			0.067
mean (SD^a^)	31.3 (5.7)	33(4.9)	
**Gestational age**			0.562
median (IQR^b^)	36 (33–39)	37 (35–38)	
**Gravidity**			0.346
1	52(43.33)	17(35.42)	
>1	68 (56.67)	31 (64.58)	
**Parity**			0.76
1	78 (65.0)	30 (62.5)	
>1	42 (35.0)	18 (37.5)	
**Smoking**			0.060
No	87(83.65)	42(95.45)	
Yes	17(16.35)	2(4.55)	
**BMI**^c^			0.352
<25	57(55.88)	27(64.29)	
≥25	45(44.12)	15(35.71)	
**BMI**^c^			0.298
median (IQR^b^)	24.4(21.5–28.3)	23.6(21.1–25.2)	
**Preeclampsia**			0.070
No	71(58.68)	36(73.47)	
Yes	50(41.32)	13(26.53)	
**Diabetes**			0.449
No	112 (93.33)	48 (97.96)	
Yes	8 (6.67)	1 (2.04)	
**CA diameter**			0.760
Median (IQR^b^)	11 (6–18)	11 (5–20)	

^a^SD-standard deviation;

^b^IQ-inter-quartile range;

^**c**^BMI-body mass index.

The incidence of the 5 minutes APGAR with <7 (11.02% vs 4.04%, OR = 3.21, 95%CI = 1.01–10.22, p = 0.047), abnormal PI (33.33% vs 16.67%, OR = 2.50, 95%CI = 1.05–5.92, p = 0.037) and BFC ≥ 1 (33.96% vs 14.49%, OR = 3.03, 95%CI = 1.25–7.30, p = 0.013) were significantly higher in neonates from singleton compared to multiple pregnancies group, while the frequency of stillbirths (10.13% vs 3.03%), although was higher in singleton compared to the multiple neonates group, reached only a marginal significant level. There were no further statistically significant associations revealed in studied outcomes between the neonatal groups (Table 2).

**Table 2:** Neonatal outcome characteristics in singleton and multiple pregnancies.

	Singleton	Multiple	P value
	(N = 121)	(N = 100)	
**Gender**			0.161
female	61(52.14)	61(61.62)	
male	56(47.86)	38(38.30)	
**Mode of delivery**			0.280
vaginal	44 (36.36)	29(29.00)	
cesarean section	76 (62.81)	71(71.00)	
termination	1(0.83)	0(0)	
**Prematurity**			0.260
≥37 weeks	61(50.41)	58(58.00)	
<37 weeks	60(49.59)	42(42.00)	
**Birth weight vs gestational age**^a^			0.621
SGA	51(42.15)	37(37.76)	
AGA	69(57.02)	61(62.24)	
LGA	1(0.83)	0 (0)	
**5 min Apgar**			**0.012**
≥7	93(78.81)	92(92.93)	
<7	13(11.02)	4(4.04)	
IUFD^b^	12(10.17)	3(3.03)	
**NICU**^c^ **admission**			0.890
no	66(55.93)	55(55.00)	
yes	52(44.07)	45(45.00)	
**PI**^d^			**0.034**
normal	32(66.67)	60(83.33)	
abnormal	16(33.33)	12(16.67)	
**BFC**^e^			**0.011**
<1	35(66.04)	59(85.51)	
≥1	18(33.96)	10(14.49)	
**Outcome**			0.057
liveborn	106(89.83)	96(96.97)	
stillborn	12(10.17)	3 (3.03)	

^a^SGA-small for gestational age; AGA-appropriate for gestational age; LGA-large for gestational age;

^b^IUFD-intrauterine fetal death;

^c^NICU-neonatal intensive care unit;

^d^PI-pulsatility index;

^e^BFC-blood flow class

In the singleton pregnancies, the median diameter of the CA was significantly smaller in mothers with preeclampsia compared to the group without preeclampsia (9mm vs 14mm, p = 0.039, respectively), whereas the multiparity (15mm vs 9mm, p = 0.005) and smoking (15mm vs 9mm, p = 0.005) in maternal groups were related to a significantly larger median diameter of CA in comparison to primiparity and non smoking groups, respectively (Table 3).

**Table 3:** Association of maternal outcomes with diameter of CA in singleton and multiple pregnancies.

	Singleton Mothers	P value	Multiple Mothers	P value
	CA mm,median(IQR)		CA mm,median(IQR)	
**Preeclampsia**		**0.039**		0.256
No	14(6–30)		13.5(4.5–20)	
Yes	9(6–12.8)		7(5–12)	
**Diabetes**		0.138		0.190
No	11 (6–19)		10.5(4.8–19.2)	
Yes	6.5(3.8–9)		35	
**Parity**		**0.005**		0.831
1	9(4.2–15)		11.5(2.8–20)	
>1	15(7–30)		10.5(5.8–17.2)	
**Gravidity**		0.198		0.837
1	9.5(6–16.2)		13(5–20)	
>1	12(6–19.5)		10(4.5–19)	
**Smoking**		**0.001**		0.204
No	9(5–16)		11.5(5–20)	
Yes	27(11–40)		5(3–7)	
**BMI**^a^		0.115		0.916
<25	13(6–19)		11(2.5–20)	
≥25	9(5–14)		10(7.5–16.5)	

^**a**^BMI-body mass index

Further, the median diameter of CAs in neonates from singleton pregnancies was significantly smaller in small for gestational age (SGA) compared to appropriate for gestational age (AGA) group (8mm vs 14mm, p = 0.00040), as well as was smaller within the group of neonates admitted to the neonatal care unit (NICU) compared to no admitted group (8mm vs 13.5mm, p = 0.028), respectively, (Table 4). No statistical significant associations with any of the studied neonatal outcomes in the group of neonates from the multiple pregnancies and the diameter of the CAs have been observed (Table 4).

**Table 4:** Association of neonatal outcomes with diameter of CA in singleton and multiple pregnancies.

	SINGLETON NEONATES	P value	MULTIPLE NEONATES	P value
	CA mm, median(IQR)		CA mm, median(IQR)	
**Gender**		0.861		0.124
female	10.5(6–17)		10(2–18)	
male	10.5(5–19.2)		15(7.8–20)	
**Mode of Delivery**		0.059		0.114
vaginal	14(7.5–30)		7(1–20)	
cesarean section	9(5–15.2)		12(7–19.5)	
termination	5		0(0)	
**Prematurity**		0.071		0.302
≥37 weeks	13(6–30)		12(6–20)	
<37 weeks	9(5.5–15)		10(5–15)	
**Birth weight vs gestational age**^a^		**0.00040**		0.412
SGA	8(4–11.5)		13(6–20)	
AGA	14(6–30)		10(5–18)	
**5 min Apgar**		0.983		0.862
≥ 7	11(6–18.2)		12(5–20)	
< 7	10(6–16)		8.5(6.2–17.5)	
IUFD^b^	14(4.2–20.5)		3(2.5–16.5)	
**NICU**^c^ **admission**		**0.028**		0.457
no	13.5(6–26.5)		12(4–18)	
yes	8(5–14.5)		10(5–30)	
**PI**^d^		0.303		0.268
normal	11(6–17)		12.5(4.2–20)	
abnormal	8(5–11.2)		6.5(5–13.2)	
**BFC**^e^		0.066		0.979
<1	11(6–17)		12(5–20)	
≥1	6.5(4.2–10)		10(6.2–19.2)	
**Outcome**		0.857		0.609
liveborn	11(6–18)		12(5–20)	
stillborn	14(4.2–20.5)		3(2.5–16.5)	

^a^AGA-appropriate for gestational age; SGA-small for gestational age; LGA-large for gestational age;

^b^IUFD-intrauterine fetal death;

^c^NICU-neonatal intensive care unit;

^d^PI-pulsatility index;

^e^BFC-blood flow class.

## Discussion

In the present study, we assessed whether the pregnancy outcome differs significantly between singleton and multiple gestations with CAs and how it is related to the diameter of the CA. The results showed no statistically significant difference in any of the investigated maternal clinical characteristics between the two studied groups. However, neonates in the multiple pregnancies with CAs demonstrated a much more favorable outcome than the singleton pregnancies with CAs, showing lower incidence of low 5-minute Apgar score, stillbirths, pathological PI and BFC ≥1.

In our institution only selected placentas according to the regional consensus indications are referred to the pathological examination. Therefore, the current study comprises a high-risk pregnancy population with a high incidence of preeclampsia. Previous reports have shown that women with multiple gestations are at two to three times higher risk for the development of hypertensive disorders of pregnancy, including preeclampsia [7–10]. However, our current data analysis from a highly selected cohort of placentas with CA, demonstrates an opposite association and shows a higher incidence of preeclampsia in singleton rather in multiple pregnancies (41.32% vs 26.53%, respectively).

It has been suggested that due to the increased fetal demand for blood in multiple gestations, elevated blood pressure might be related to a better fetal oxygen and nutrient supply, to have protective effects against low 5-minute Apgar score and to be beneficial to fetal survival in multiple compared to singleton pregnancies [7,10]. It has also been reported that hypertension might reduce the risk for fetal and neonatal mortality in premature cases of multiple pregnancies, while in singleton pregnancies hypertension is related to adverse neonatal outcome [7].

Further, due to a greater demand for blood from multiple fetuses, Luo et al speculated that pregnancy-induced hypertension might have more hypoxia related placental pathologic changes in singleton rather in multiple pregnancies [10]. In a previous study, we have demonstrated that singleton placentas with CA were affected by several hypoxia-related placental changes, whereas in multiple pregnancies these changes were not present [2]. Thus, we hypothesized that multiple pregnancies *per se* with increased demand of oxygen are associated with placental tissue hypoxia [2]. Hence, our observation that abnormal blood flow velocity waveforms in the umbilical artery occurred more often in the singleton pregnancies may support such a speculation. Therefore, in singleton pregnancies CA seems to be associated with an increased placental resistance, while in multiple pregnancies such phenomenon is less common when evaluated by velocimetry in umbilical artery and is not related to the size of the CA.

Based on the current study we provide evidence that CAs with smaller diameter are associated to preeclampsia and small for gestational age (SGA) neonates in singleton pregnancies; suggesting that, small CAs could be considered as preeclampsia induced hypoxic vascular hyperplasias, rather than true tumors. Noteworthy, hypoxia from reduced placental perfusion, have shown to stimulate circulating levels of angiogenic/antiangiogenic factors of placental origin including the soluble fms-like tyrosine kinase 1 (sFlt-1) molecule [11]. The possibility of chronic hypo-perfusion in the placentas of multiple pregnancies could also be hypothesized to explain the increased sFlt-1 observed in multiple compared to singleton pregnancies, while multiple pregnancies may share similar pathological mechanisms hypothesized to occur in preeclampsia [11]. In particular, analysis of angiogenic factors throughout the pregnancy has shown elevated maternal concentrations of sFlt-1 and sFlt-1/PIGF ratio in multiple compared to singleton pregnancies without preeclampsia [12]. The latter might indicate a shared pathological pattern and/or to reflect an increased hypoxic environment in utero in multiple pregnancies as well as in preeclampsia [11].

Interestingly, van Gemert et al recently published on a hypothesis that the use of umbilical/chorionic venous flows could identify CA that could have serious circulatory consequences on the fetus [13]. The present study includes too few cases with big CAs to accurately study this hypothesis. However, since in many of cases with large CA one dominating vessel is possible to identify it is highly likely that this method is of clinical relevance when evaluating CA with ultrasound before birth. Also, it needs to be taken into consideration that the vasculature anatomy and flow physiology is better evaluated by in vivo ultrasound studies rather than after birth.

In conclusion, our study showed a much more favorable pregnancy outcome in multiple gestations compared to the singleton gestations with CAs, which might reflect an adaptive mechanism for relative intrauterine hypoxia per se in this group. We also demonstrated that singleton placentas were affected by hypoxia related changes and singleton pregnancies were associated with an increased rate of adverse neonatal outcome. Further, the frequency of preeclampsia was elevated in both studied groups, although it was more common in the group of singleton pregnancies. Noticeably, a high incidence of preeclampsia in cohort of placental CAs might lead to a possible recognition of CAs as potential morphologic indicator of placental hypoxia, which could be an invaluable contribution in clinical placental diagnostics. To best of our knowledge the current study is the largest cohort study so far investigating the clinical characteristics of patients with placental CAs in singleton and multiple pregnancies, as well as assessing the related neonatal outcomes; hence providing valuable insights into the clinical approach of placental chorangiomas.
